# Rosinidin Attenuates Lipopolysaccharide-Induced Memory Impairment in Rats: Possible Mechanisms of Action Include Antioxidant and Anti-Inflammatory Effects

**DOI:** 10.3390/biom11121747

**Published:** 2021-11-23

**Authors:** Sultan Alshehri, Syed Sarim Imam

**Affiliations:** Department of Pharmaceutics, College of Pharmacy, King Saud University, P.O. Box 2457, Riyadh 11451, Saudi Arabia; salshehri1@ksu.edu.sa

**Keywords:** acetylcholinesterase, anthocyanidin, flavonoids, neuroprotective

## Abstract

The investigation aimed to evaluate the favourable effects of rosinidin in lipopolysaccharide (LPS)-induced learning and memory impairment in rats. Adult Wistar rats (150–200 g) were segregated equally into four different groups and treated as below: Group 1 (normal) and Group 2 (LPS control) were administered orally with 3 mL of 0.5% SCMC (vehicle); Group 3 and Group 4 were test groups and orally administered with rosinidin lower dose (10 mg/kg) and higher dose 20 mg/kg. Daily, 1 h post-offer mentioned treatments, Group 1 animals were injected with normal saline (i.p.) and groups 2–4 were treated with 1 mg/kg/day of LPS. This treatment schedule was followed daily for 7 days. During the treatment, schedule rats were evaluated for spontaneous locomotor activity, memory, and learning abilities. The biochemical assessment was carried out of acetylcholine esterase (AChE), endogenous antioxidants (GSH, SOD, GPx, and catalase), oxidative stress marker MDA, neuroinflammatory markers (IL-6, IL-1β, TNF-α, and NF-κB), and BDNF. LPS-induced reduced spontaneous locomotor activity and memory impairment in the animals. Moreover, LPS reduced GSH, SOD, GPx, and catalase levels; altered activities of AChE; elevated levels of MDA, IL-6, IL-1β, TNF-α, and NF-κB; and attenuated the levels of BDNF in brain tissue. Administration of rosinidin to LPS-treated animals significantly reduced LPS-induced neurobehavioral impairments, oxidative stress, neuroinflammatory markers, and reversed the Ach enzyme activities and BDNF levels towards normal. Results demonstrated that rosinidin attenuates the effects of LPS on learning memory in rats.

## 1. Introduction

Neurodegenerative diseases are characterized by inflammation of the nervous system. As a result of neuroinflammation and mitochondrial dysfunction, reactive oxygen (ROS) and nitrogen (RNS) species are delivered at extreme levels [1,2,3]. The cell wall component lipopolysaccharide (LPS) that is found in Gram-negative bacteria is often used against animals as a cause of inflammation in the nervous system [1,2]. Systemic LPS has been identified as a pathogen-associated molecule pattern by higher vertebrates.

By binding to immune cells, LPS activates nuclear factor κB (NFκB) to increase the expression of tumour necrosis factor (TNF-α), interleukin-6 (IL-6), and interleukin-1β (IL-1β). Following the release of cytokines, microglia and macrophages in the central nervous system (CNS) also produce the same cytokines, targeting neuronal substrates and inducing inflammation within neurons [1,2]. A rapid inflammatory response caused by LPS produces relatively high levels of peroxides and ROS in the CNS [1,2]. Ultimately, oxidative stress-mediated pathology results when levels of peroxides and ROS exceed endogenous antioxidant defence [1,2]. Lipid peroxidation targets polyunsaturated fatty acids in the brain [4,5]. Moreover, the brain has few antioxidant defence mechanisms, therefore, it is highly susceptible to oxidative damage [4,5,6]. Additionally, LPS results in behavioural abnormalities such as cognitive abnormalities and dementia [1,2,5]. It is possible to lower the prevalence of neurodegenerative diseases by reducing neuroinflammation and oxidative stress early [1,2]. Recent studies have proven that antioxidants and anti-inflammatory agents are beneficial for treating various CNS pathologies, including inflammation and oxidative stress induced by LPS [1,2,5,7].

In neurodegenerative diseases, flavonoids inhibit inflammatory mediators, activate antioxidant enzymes, suppress lipid peroxidation, and modulate gene expression [8]. Many flavonoids have been reported to possess neuroprotective actions in different models of neurodegenerative diseases [9,10].

The fruits and flowers of higher plants contain red-blue water-soluble flavonoids anthocyanin and its sugar-free counterpart anthocyanidin. Both anthocyanin and anthocyanidin are used as a colourant in various food and as pharmaceutical ingredients [11]. Moreover, anthocyanin and anthocyanidin have potential health benefits [11,12]. Anthocyanins and their metabolites were studied for neuroprotective actions in various neurodegenerative diseases [13]. Anthocyanin showed beneficial effects in depression by increasing neurotransmitter monoamine and brain-derived neurotrophic factor (BDNF) expression up-regulation [14].

**Figure 1:**
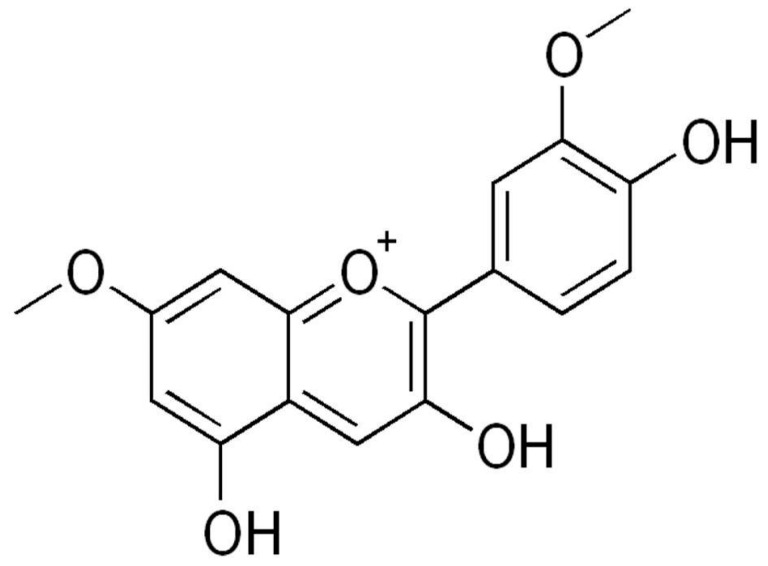
Chemical structure of rosinidin.

Rosinidin is a flavonoid (anthocyanidin) found as a pigment in flowers like *Catharanthus roseus* and *Primula rosea*. Rosinidin (Figure 1) consists of benzopyrylium with hydroxy substituents at positions 3 and 5, methoxy substituents at positions 7, and a 4-hydroxy-3-methoxyphenyl substitution at position 2.

In silico enzymatic target, studies revealed that rosinidin has necessary structural properties and pharmacological actions and it has potential to be a drug candidate for neurodegenerative treatment [8]. Molecular docking studies showed that rosinidin has good neuroprotective action against Parkinson’s disease [8]. In light of the above data, the study was conducted to evaluate the effectiveness of rosinidin in LPS-induced memory impairment in rats.

## 2. Methodology

### 2.1. Chemicals

Rosinidin and LPS were procured from Sigma Aldrich (St. Louis, MO, USA). The analytical kits for interleukin-6 (IL-6), interleukin-1β (IL-1β), tumour necrosis factor alpha (TNF-α), nuclear factor kappa (NF-κB), and brain-derived neurotrophic factor (BDNF) were measured by using commercially available rat enzyme-linked immunosorbent assay kit, India (Modern Lab, M.S., Indore, India). The experiment was performed using high-quality reagents and chemicals.

### 2.2. Animals

Wistar rats (200–240 g) were acclimatized to laboratory conditions. They were free to access food and water. Animal Ethics Committee of the Institution approved the protocol, which followed guidelines of the CPCSEA, Government of India.

### 2.3. Acute Oral Toxicity Studies

The acute oral toxicity study (LD50) of rosinidin was performed as per the guidelines set by the Organization for Economic Cooperation and Development (OECD), ANNEX-423 [15,16].

### 2.4. Experimental

Rosinidin was diluted with 0.5% sodium CMC solution and provided to experimental animals orally for 07 days. To induce neuroinflammation and memory impairment in rats, 1 mg/kg of LPS was given intra-peritoneally after freshly diluting with saline (pH 7.4) [1,2,5].

**Figure 2:**
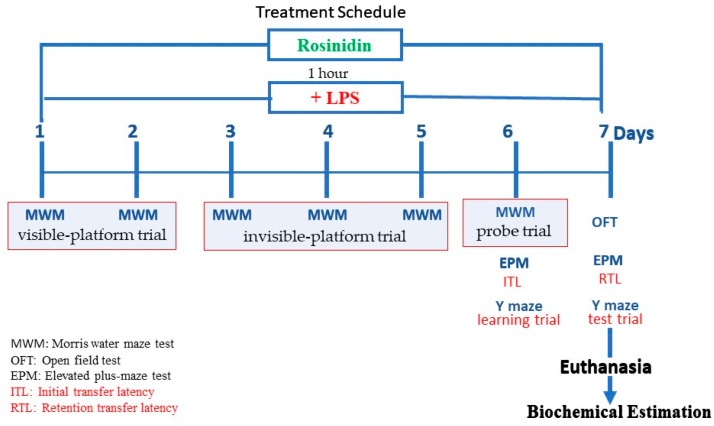
Schematic diagram of experimental protocol.

Total 24 rats (n = 6) were equally segregated into four different groups and given the following treatments: Groups I-normal and II-LPS control groups were treated with 3 mL/kg of 0.5% sodium CMC. The test groups III-lower dose and IV-higher dose received 10 and 20 mg/kg (p.o.) of rosinidin suspension in 0.5% SCMC. Every day 1 h post above oral treatments, Group I was treated with 3 mL/kg (i.p.) of normal saline/day, and 1 mg/kg/day of LPS was injected (i.p.) to groups II–IV. All the above-mentioned treatments were given daily for 7 days. During the treatment schedule, 2 h post LPS treatment behavioural tests were conducted for animals. On the 7th day after behavioural tests animals were sacrificed and brains were removed for biochemical tests [1,2,5]. The experimental protocol is schematically represented in Figure 2.

### 2.5. Behavioural Parameters

#### 2.5.1. Open Field Test

The open field consists of a large cubic wooden box with dimensions of 1.2 m long × 1.2 m wide × 50 cm high, its floor divided into 16 squares. The 12 squares along the walls were considered the peripheral squares and the remaining four squares were central. Individual rats were placed in open fields for five minutes each and climbing, rearing, and line crossings of the animal were recorded. When an animal leans against a wall with its front paws it is considered as climbing; when both front paws were lifted from the floor it is counted as rearing; and taking all four paws away from one square and placing them into another square is line crossing. Crossings between the central squares and the peripheral squares were counted separately [17,18].

#### 2.5.2. Elevated Plus Maze (EPM) Test

EPM is made up of two open (50 × 10 cm) and closed arms of identical proportions and a 40 cm sidewall. The central square (10 cm^2^) joins the arms of EPM. On day 6, memory acquisition was measured by placing the animal at the terminal part of one open arm, faced towards the centre square. Initial transfer latency (ITL) was measured as the duration required for a rat to enter in one of the closed arms from an open arm. If any animal fails to enter closed arm in 2 min, the rat was gently assisted to enter in one closed arm, allowed to explore the closed arm for 10 s and recorded 120 s as its ITL. On day 7, the retention transfer latency (RTL) was measured following the same procedure as ITL [3,4,7].

#### 2.5.3. Y Maze Test

The Y maze is made up of a triangular central region connected to three-compartment arms made from black-painted Plexiglas. On the 6th day of the treatment schedule, a 2 h post LPS treatment learning trial was conducted on the animals. During the learning trial, each rat was exposed to the Y maze apparatus and animals were allowed 5 min to move freely in the compartments. There were two compartments in the maze where the electric shocks (2 Hz, 10 V for 125 ms) were passed through stainless steel rods. To avoid the electric shock, animals would try to find an electric shock free area and enter a shock-free compartment. An animal was allowed to remain in shock-free space for 30 s and training was ended. The time it took the animal to enter the shock-free compartment after the electric shock started was noted. On day 7, 2 h post LPS treatment, similar to the trial day, a Y maze test was performed and the time it took for the animal to enter into a shock-free compartment was recorded. The difference in latency from the 6th and 7th days was recorded [1,2,19,20].

#### 2.5.4. Morris Water Maze (MWM) Test

In this test, following five consecutive days of training, a probe test was administered on the sixth day. Tests were conducted in a circular pool (120 cm diameter, 50 cm height), filled with 30 cm of water (25 ± 1 °C). An immobilized white platform (9 cm in diameter) was placed 1 cm under the surface of the water during training. A test was conducted with rats immersed in maze water for 90 s to seek platforms. The visible-platform trial was conducted on days 1–2; during this a flag (5 cm high) was displayed on the platform to make it visible. The invisible-platform trial was conducted on days 3–5, and during this there was no flag was displayed on the platform. On day 6, the probe trial was conducted without a platform [1,2,21].

### 2.6. Biochemical Parameters

#### 2.6.1. Brain Tissue Homogenisation

Animals were decapitated, the brain was separated and cleaned using ice-cold isotonic saline. In phosphate buffer (0.1 M, pH 7.4, ice-cold), brain samples were homogenised. The homogenate was centrifuged and performed the biochemical analysis by using supernatant [3,4,7].

#### 2.6.2. Acetylcholinesterase (AchE) Activity

A protocol similar to that provided by Ellman et al. (1961) was followed to determine AchE activity represented as μM/mg protein [22].

### 2.7. Oxidative Stress Parameters

Malondialdehyde (MDA) was estimated in brain homogenate using the Wills method. The MDA level was represented as nM/mg protein [23]. The reduced glutathione (GSH) was quantified as per a previously described method by Ellman [24]. Using the Misra and Frodvich method, superoxide dismutase (SOD) was determined. The SOD activity was expressed as a percentage of control [25]. According to the method given in Razygraev et al., 2018, glutathione peroxidase (GPx) activity was measured [26]. To estimate the Catalase activity, 0.1 mL of supernatant was added to 1.9 mL phosphate buffer (pH 7.0, 50 mM) in the cuvette. Then, 1.0 mL of freshly prepared H_2_O_2_ (30 mM) was added to initiate the reaction. The catalase activity was represented as μM/H_2_O_2_ decomposed/min [27].

### 2.8. Neuroinflammatory Markers and BDNF

The IL-6, IL-1β, TNF-α, NF-κB, and BDNF were quantified by immunoassay kit. The concentrations of markers were calculated and expressed in pg/mL protein.

### 2.9. Statistical Analysis

Statistical analysis was performed through Prism software. The data are presented as the mean ± S.E.M. One way ANOVA was followed by Tukey’s test, and the significance level was set at *p* < 0.05.

## 3. Results

Acute oral toxicity studies revealed that Rosinidin was safe at a maximum oral dose of 200 mg/kg b.w. in mice. No lethality or toxic reactions were observed in 14 days period. Based on acute oral toxicity studies data 1/20th and 1/10th dose, i.e., 10 mg/kg and 20 mg/kg, were selected for further study.

### 3.1. Behavioural Parameters

#### 3.1.1. Open Field Test

**Figure 3:**
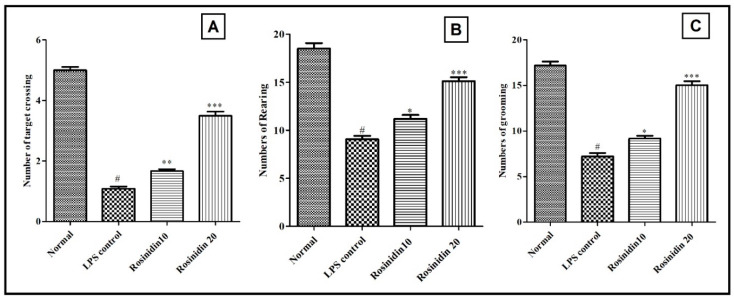
Effect of rosinidin on spontaneous locomotory activities in open field test on LPS-treated rats. (**A**) Target crossing, (**B**) rearing, and (**C**) grooming. Values are expressed as mean ± S.E.M. (n = 6). # *p* < 0.05 vs. normal control rats and * *p* < 0.05; ** *p* < 0.01; and *** *p* < 0.001 vs. LPS control rats. One-way ANOVA followed by Tukey’s post hoc test.

An open field was used to measure spontaneous locomotor activity of the animals. The number of rears (9.05 ± 0.35), grooming (7.22 ± 0.36), and line crossings (1.08 ± 0.07) were significantly decreased following LPS treatment when compared with normal control (18.50 ± 0.56, 17.19 ± 0.43 and 5.0 ± 0.11, respectively). In comparison with LPS control group, rosinidin (10 and 20 mg/kg) pretreatment dose dependently improves LPS-induced decrease in line crossings (1.66 ± 0.06 and 3.49 ± 0.14) and climbs rear up (11.19 ± 0.39 and 15.19 ± 0.41). The results of open field test are shown in Figure 3.

#### 3.1.2. EPM Test

**Figure 4:**
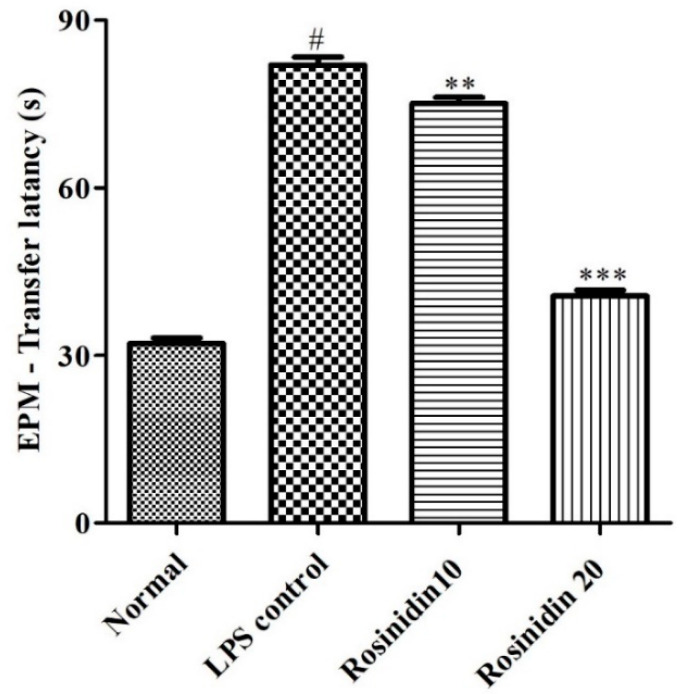
Effect of rosinidin on memory and learning abilities of animals estimated using EPM test on LPS-treated rats. Values are expressed in mean ± S.E.M. (n = 6). # *p* < 0.05 vs. normal control rats and ** *p* < 0.01; and *** *p* < 0.001 vs. LPS control rats. One-way ANOVA followed by Tukey’s post hoc test.

Figure 4 represents the results of memory and learning abilities of animals estimated using EPM test. LPS resulted in considerably (*p* < 0.05) increased transfer latency in animals (82 ± 1.41). Treatment of lower (75.17 ± 1.01) and higher (40.67 ± 1.05) doses of rosinidin dose dependently restored the transfer latency in the animals. The results were statistically significant (*p* < 0.01), and correlated with LPS-control animals.

#### 3.1.3. Y Maze Test

**Figure 5:**
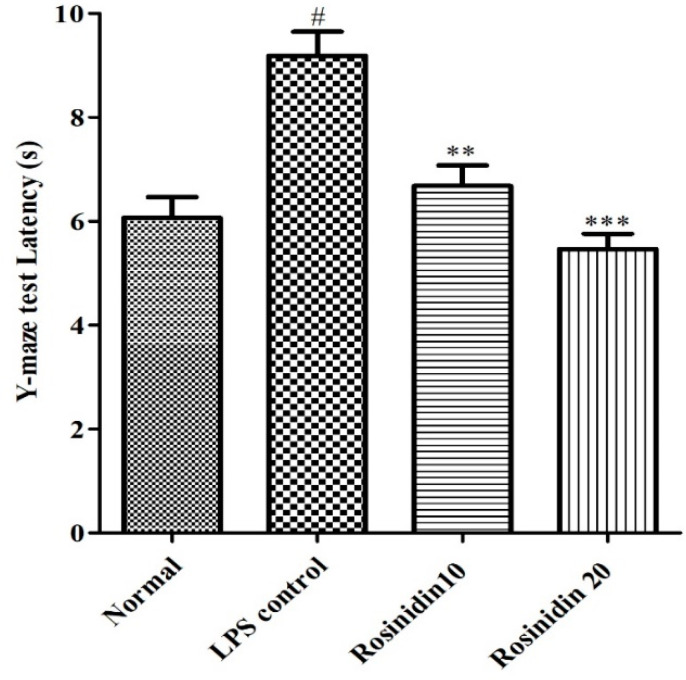
Effect of rosinidin on Y-maze test on LPS-treated rats. Values are expressed in mean ± S.E.M. (n = 8). # *p* < 0.05 vs. normal control rats and ** *p* < 0.01; and *** *p* < 0.001 vs. LPS control rats. One-way ANOVA followed by Tukey’s post hoc test.

Treatment with LPS (9.18 ± 0.46) increased the latency of transfer (*p* < 0.05) compared to the normal animals (6.06 ± 0.39). Administration of rosinidin to LPS treated rats dose dependently restored the transfer latency (6.67 ± 0.39 and 5.46 ± 0.46, respectively), compared to LPS-control animals (*p* < 0.01). The result of the Y maze test is represented in Figure 5.

#### 3.1.4. MWM Test

**Figure 6:**
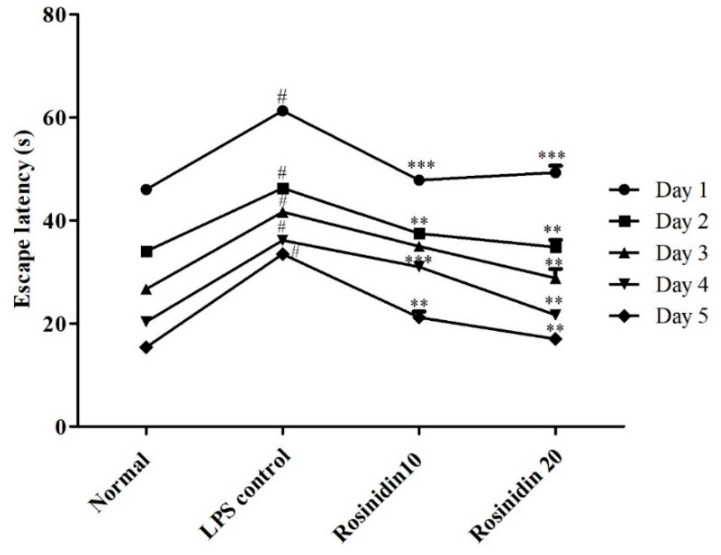
Effect of rosinidin on Morris water maze test in LPS-treated rats. Values are expressed in mean ± S.E.M. (n = 6). # *p* < 0.05 vs. normal control rats and ** *p* < 0.01; and *** *p* < 0.001 vs. LPS control rats. Two-way ANOVA followed by Bonferroni post hoc test.

Administration of LPS significantly (*p* < 0.05) increased the escape latency in MWM test in all intervals. Administration of rosinidin to LPS treated animals significantly decreased the escape latency in animals. The values were statistically significant at both lower and higher doses of rosinidin treatment (*p* < 0.001). The detailed result of MWM test is shown in Figure 6.

### 3.2. Biochemical Parameters

#### AchE Activity

**Figure 7:**
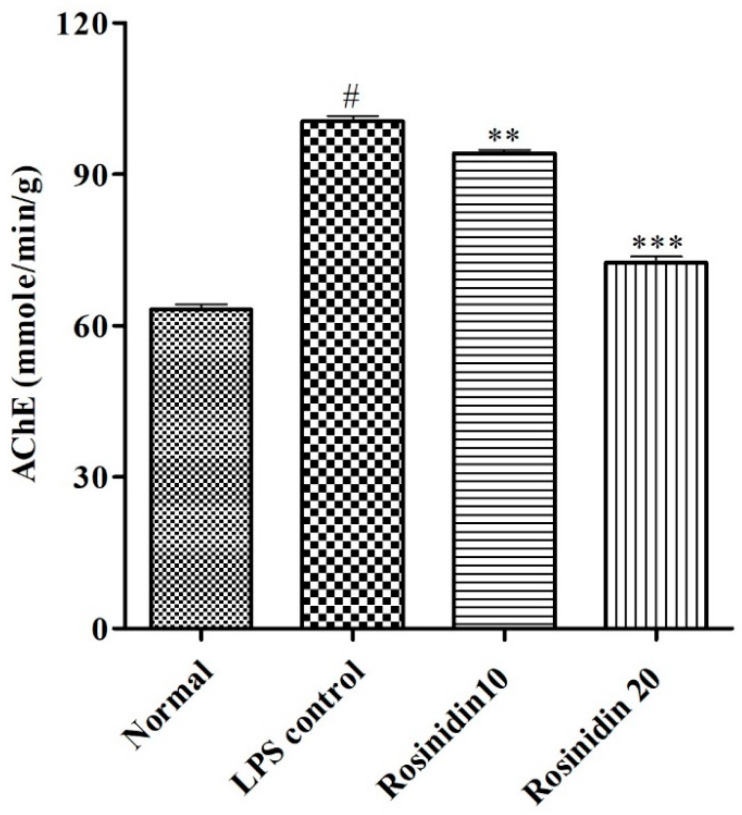
Effect of rosinidin on acetylcholinesterase activity in LPS-treated rats. Values are expressed in mean ± S.E.M. (n = 6). # *p* < 0.05 vs. normal control rats and ** *p* < 0.01 and *** *p* < 0.001 vs. LPS control rats. One-way ANOVA followed by Tukey’s post hoc test.

AchE level was significantly (*p* < 0.05) increased in LPS control group (100.5 ± 1.08) when correlated to normal control (63.17 ± 1.04). Treatment with rosinidin to LPS treated animals significantly (*p* < 0.01) reduced the amount of AchE (94.17 ± 0.74 and 72.50 ± 1.17) compared to LPS control animals. The result of AchE estimation is represented in Figure 7.

### 3.3. Oxidative Stress Parameters

#### 3.3.1. MDA Levels

**Figure 8:**
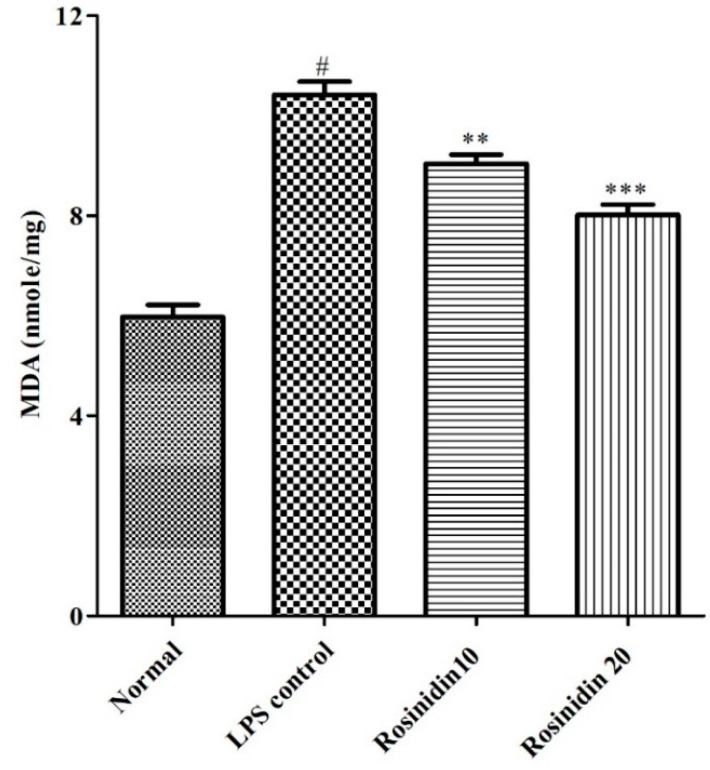
Effect of rosinidin on malondialdehyde levels in LPS-treated rats. Values are expressed as mean ± S.E.M. (n = 6). # *p* < 0.05 vs. normal control rats and ** *p* < 0.01 and *** *p* < 0.001 vs. LPS control rats. One-way ANOVA followed by Tukey’s post hoc test.

Animals treated with LPS developed oxidative stress in their brains. The elevated MDA level (10.42 ± 0.26) was observed in LPS treated animals compared to normal control animals (5.97 ± 0.24). Rosinidin treatment dose dependently attenuated (9.04 ± 0.18 and 8.01 ± 0.20) the increased levels of MDA towards normal compared to LPS control group (*p* < 0.01 and *p* < 0.001). The result of MDA levels is shown in Figure 8.

#### 3.3.2. Endogenous Antioxidant Status

**Figure 9:**
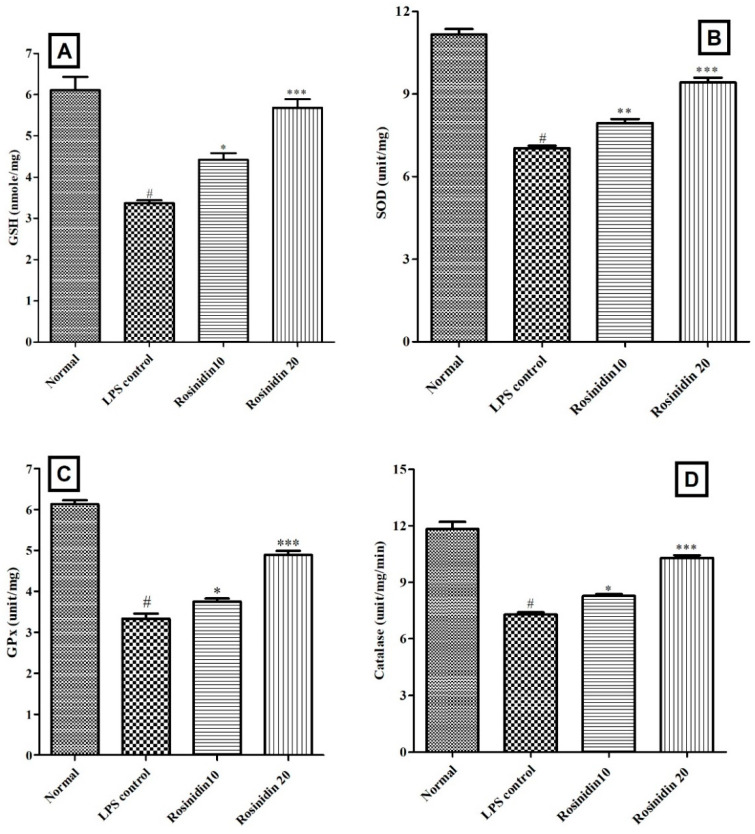
Effect of rosinidin on endogenous antioxidant status in LPS-treated rats. (**A**) Reduced glutathione, (**B**) superoxide dismutase, (**C**) glutathione peroxidase, and (**D**) catalase. Values are expressed in mean ± S.E.M. (n = 6). # *p* < 0.05 vs. normal control rats and * *p* < 0.05, ** *p* < 0.01 and *** *p* < 0.001 vs. LPS control rats. One-way ANOVA followed by Tukey’s post hoc test.

Administration of LPS disturbed the endogenous antioxidants levels. Considerably (*p* < 0.05) reduced SOD (7.03 ± 0.09), GSH (3.36 ± 0.07), GPx (3.33 ± 0.12), and catalase (7.29 ± 0.11) levels were observed in LPS control animals correlated to normal control. Treatment with rosinidin (10 and 20 mg/kg) to LPS injected animals improved the endogenous antioxidant status in the treated animals in dose dependent manner, GSH (4.41 ± 0.16 and 5.68 ± 0.20, *p* < 0.05), SOD (7.95 ± 0.14 and 9.42 ± 0.16, *p* < 0.01), GPx (3.75 ± 0.07 and 4.89 ± 0.10, *p* < 0.05), and catalase (8.27 ± 0.09 and 10.29 ± 0.14, *p* < 0.05) levels towards normal. The endogenous antioxidant status results are shown in Figure 9.

### 3.4. Neuroinflammatory Markers

**Figure 10:**
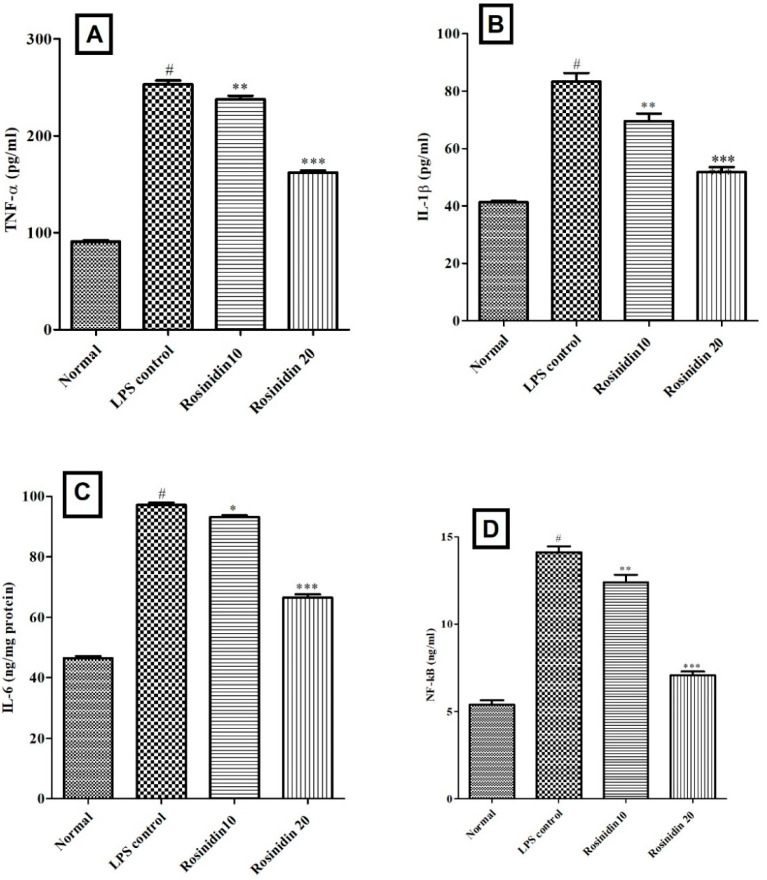
Effect of rosinidin on endogenous antioxidant status in LPS-treated rats. (**A**) tumour necrosis factor (TNF-α), (**B**) interleukin-1 β (IL-1β), (**C**) interleukin-6 (IL-6) and (**D**) nuclear factor κB (NFκB). Values are expressed in mean ± S.E.M. (n = 6). # *p* < 0.05 vs. normal control rats and * *p* < 0.05, ** *p* < 0.01, and *** *p* < 0.001 vs. LPS control rats. One-way ANOVA followed by Tukey’s post hoc test.

The levels of IL-1β (83.33 ± 3.0), IL-6 (97.17 ± 0.75), TNF-α (253 ± 4.0), and NF-κB (14.12 ± 0.33) were significantly (*p* < 0.05) high in LPS treated animals when correlated to normal control animals. Rosinidin attenuated the levels of TNF-α (237.7 ± 3.65 and 165 ± 2.22, *p* < 0.01), IL-1β (69.67 ± 2.53 and 51.83 ± 1.74, *p* < 0.01), IL-6 (93.14 ± 0.64 and 66.43 ± 1.13, *p* < 0.05) and NF-κB (12.40 ± 0.42 and 7.06 ± 0.20, *p* < 0.01), significantly. The TNF-α, IL-1β, IL-6 and NF-κB results are represented in Figure 10.

### 3.5. BDNF

**Figure 11:**
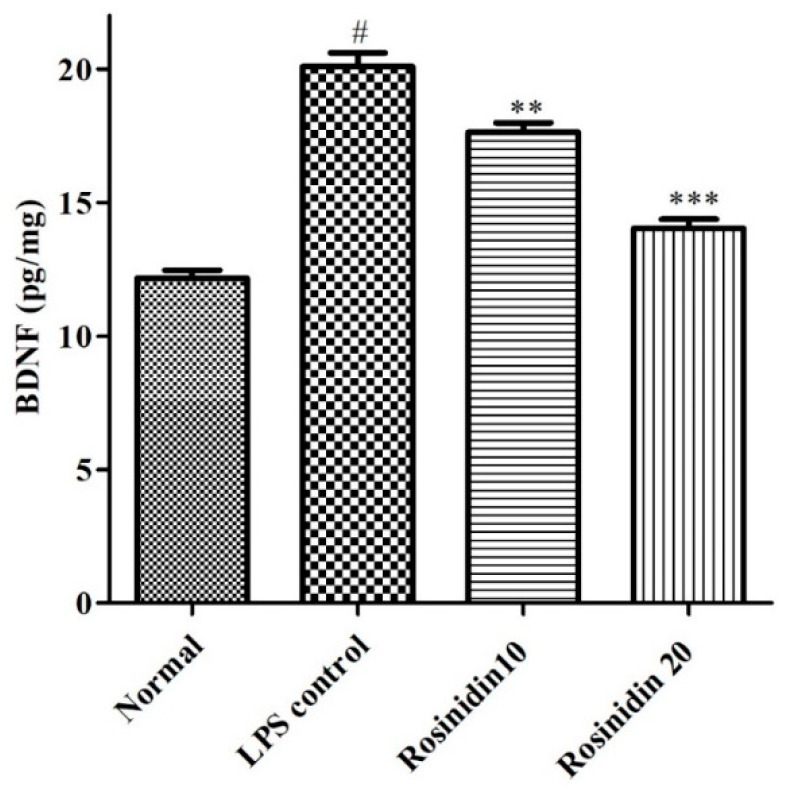
Effect of rosinidin on BDNF levels in LPS-treated rats. Values are expressed in mean ± S.E.M. (n = 6). # *p* < 0.05 vs. normal control rats and ** *p* < 0.01 and *** *p* < 0.001 vs. LPS control rats. One-way ANOVA followed by Tukey’s post hoc test.

The levels of BDNF were decreased (*p* < 0.05) considerably in LPS treated animals (12.17 ± 0.30) compared to normal control (20.10 ± 0.51). Administration of rosinidin to LPS treated animals dose dependently restored (14.04 ± 0.34 and 17.63 ± 0.35) the BDNF levels correlated to LPS group. The values were statistically significant (*p* < 0.05) vs. LPS control group. The result of BDNF levels is represented in Figure 11.

## 4. Discussion

LPS-induced memory impairments and behavioural abnormalities were evidenced by decreased spontaneous locomotor activity, spatial learning, and memory. These symptoms were attributed to elevated AchE, oxidative stress, neuroinflammatory markers, and BDNF levels in the brain tissues. On the other hand, treatment with rosinidin improved the LPS-induced behavioural and biochemical alteration. Rosinidin dose dependently improved the spontaneous locomotor activity, spatial learning, and memory and AchE changes induced by LPS. In addition, rosinidin restored the endogenous antioxidant status and decreased the neuroinflammatory markers and BDNF levels in the rat brain.

In animal psychological studies, the open field test is utilized most extensively to assess animal behaviour [28]. Behavioural information is gathered from open field test, such as general ambulatory ability and information about the emotional state of the animals [28]. In the present study, this test is used to assess spontaneous locomotory movements in LPS-treated animals. Administration of LPS resulted in a significantly decreased number of rears, grooming, and line crossings. These behavioural changes indicate the affected spontaneous locomotor activity of the animals. Motor output, exploratory drive, fear-related behaviour, sickness, circadian cycle, and a variety of other factors can influence spontaneous locomotion [29]. Treatment with rosinidin reversed the LPS-induced abnormalities in spontaneous locomotory movements in LPS treated animals, which indicates its protective action against LPS induced locomotor abnormalities.

LPS is known to cause cognitive abnormalities, dementia, decreased learning abilities, and impairment in memory [1,2,5]. The present study results well support the above findings. Administration of LPS resulted in decreased learning abilities and memory loss in the animals and it is evident by increased latency in EPM, MWM, and Y maze test of the animals. Rosinidin treatment to the LPS treated animals improved learning abilities and restored memory in the animals. These effects show the protective action of rosinidin against LPS induced abnormalities.

Moreover, treatment with rosinidin dose dependently ameliorated the LPS-induced increased activity of AChE, the key enzyme that catalyses hydrolysis of acetylcholine (Ach). Reduced Achin CNS is responsible for cognitive deficits [30]. This indicated that rosinidin restores the memory of LPS-treated animals by inhibiting AChE and ultimately by improving the Ach levels in CNS.

As stated above, the acute inflammatory response caused by LPS produces increased levels of peroxides and ROS in the CNS [1,2]. The present study data well support the above findings. Administration of LPS increased the levels of MDA and disrupted the endogenous antioxidant levels, which was evident by decreased SOD, GSH, GPx, and catalase in LPS administered animals. Treatment with rosinidin in LPS-treated rats attenuated the LPS-induced depletion of endogenous antioxidant levels and oxidative stress in the animals, which indicates the antioxidant property of rosinidin against LPS.

Inflammation plays an important role in neurodegeneration [31]. Researchers have found that patients with neurodegenerative disease display higher concentrations of IL-6,TNF-α, IL-1β, and NF-κB [31]. In the present study, LPS induced levels of inflammatory markers. Administration of rosinidin attenuated LPS-induced IL-6, TNF-α, IL-1β, and NF-κB. This indicates the anti-inflammatory properties of rosinidin against LPS-induced inflammation of neurons in rats.

BDNF, a pleiotropic protein, serves as a neurotransmitter modulator and is involved in learning and memory-related abilities [32]. Several areas of the nervous system require BDNF for their normal development [33]. BDNF is essential for the regeneration of the peripheral nerve and its myelination after nerve damage [32,34]. A link exists between lower BDNF gene expression and decreased protein levels in depressed patients [35]. Moreover, it is reported that lower BDNF levels are associated with neurodegenerative diseases [33]. Treatment with LPS decreased the levels of BDNF in brain tissue, which indicates the neuronal insults in LPS treated animals. On the other hand, treatment with rosinidin in LPS treated animals restored the BDNF levels towards normal, which indicated the action of rosinidin against LPS induced neurotoxicity.

## 5. Conclusions

The present study demonstrates that rosinidin mitigates the behavioural and biochemical abnormalities caused by LPS in rats by attenuating inflammatory response, free radical damage, and normalizing BDNF levels. The antioxidant and anti-inflammatory actions of rosinidin may be accountable for the beneficial effect. Treatment with rosinidin significantly reversed the LPS-induced reduction in spontaneous locomotor activity and memory impairment in the tested animals. LPS reduced GSH, SOD, GPx, and catalase levels; altered activities of AChE; elevated levels of MDA, IL-6, IL-1β, TNF-α, and NF-κB; and attenuated the levels of BDNF in brain tissue. The administration of rosinidin to LPS-treated animals significantly reduced LPS-induced neurobehavioral impairments, oxidative stress, neuroinflammatory markers, and reversed the Ach enzyme activities and BDNF levels towards normal. However, future research is warranted to assess the effect in the treatment of neurodegenerative disease in humans.

## Data Availability

Not applicable.
